# Completely Fluoroless, “Apron-Less” Approach to Supraventricular Tachycardia Ablation Compared to Traditional Fluoroscopy Guided Ablation: Feasibility, Safety and Clinical Outcomes

**DOI:** 10.3390/jcm14197076

**Published:** 2025-10-07

**Authors:** Andrija Nekic, Vedran Pasara, Ivan Prepolec, Ana Bilic-Pavlinovic, Ana Cala, Domagoj Kardum, Zvonimir Katic, Borka Pezo-Nikolic, Davor Puljevic, Davor Milicic, Vedran Velagic

**Affiliations:** 1 Department for Cardiovascular Diseases, University Hospital Center Zagreb, 10000 Zagreb, Croatiadomagojkardum@yahoo.com (D.K.); katiczvone3@gmail.com (Z.K.); borkapezo@yahoo.com (B.P.-N.); dpuljevic@gmail.com (D.P.); davormilicic01@gmail.com (D.M.); vvelagic@gmail.com (V.V.); 2 School of Medicine, University of Zagreb, 10000 Zagreb, Croatia; anaabilic56@gmail.com (A.B.-P.); anacala209@gmail.com (A.C.)

**Keywords:** arrhythmia, interventional electrophysiology, cardiac ablation, supraventricular tachycardia, zero fluoroscopy

## Abstract

**Background:** Catheter ablation is an established first-line treatment for supraventricular tachycardia (SVT). Traditionally, these procedures have been performed under fluoroscopic guidance. The objective of this study was to demonstrate the feasibility and safety of a completely fluoroless approach, as well as to evaluate clinical outcomes. **Methods:** This retrospective, single-center analysis included two patient cohorts, comprising a total of 400 patients: 200 in the main (fluoroless) group and 200 in the control (fluoroscopy) group. In the main group, ablation was performed using a 3D mapping system and intracardiac echocardiography (ICE) without the use of fluoroscopy and lead aprons. In the control group, procedures were conducted under fluoroscopic guidance. The primary outcomes were feasibility and safety. Secondary outcomes included acute procedural success, defined as non-inducibility of tachycardia, and chronic success, measured as arrhythmia recurrence-free survival during a 6-month follow-up period. **Results:** Completely fluoroless ablation was successfully performed in all patients in the main group (100%). Procedures were shorter in the fluoroless group (59.0 ± 25.8 min vs. 72.7 ± 34.0 min, *p* < 0.001), with no difference in acute success rates (100% vs. 100%). No serious complications occurred in the main group, compared to one event in the control group (0 vs. 1.0, *p* = 0.313). Arrhythmia recurrence rates during follow-up were similar between groups (HR: 0.877, 95% CI: 0.367–2.097, *p* = 0.769). **Conclusions:** A completely fluoroless, “apron-less” approach to SVT ablation is feasible, with complication and success rates comparable to the traditional fluoroscopy-guided approach.

## 1. Introduction

Supraventricular tachycardias (SVTs) are often symptomatic and require treatment. Catheter ablation has been established as the first-line treatment for these arrhythmias [1], using either radiofrequency (RF) or cryoenergy catheters. Traditionally, cardiac ablation procedures have been performed with the use of fluoroscopy. However, ionizing radiation is associated with detrimental stochastic effects (elevated risk of fatal and non-fatal cancers) and deterministic effects (immediate dose-dependent cellular damage), including during invasive electrophysiology [2,3]. Therefore, reducing exposure to ionizing radiation has clear benefits for both staff and patients.

The development of electroanatomical mapping systems (EAMS) has enabled the reduction and elimination of fluoroscopy, and the use of intracardiac echocardiography (ICE) has further facilitated the minimal fluoroscopy (MF) and zero fluoroscopy (ZF) approaches. These procedures have generally been shown to be non-inferior to the conventional fluoroscopy approach in terms of acute success and complication rates [4,5,6,7,8,9,10,11]. Moreover, a recent meta-analysis [12] comparing ZF/MF approach to the conventional approach reports comparable outcomes in terms of feasibility, complications and both short and long-term success rates. The objective of this study was to demonstrate the feasibility and safety of completely fluoroless, zero-fluoro (ZF), apron-less ablation procedures for supraventricular tachycardias (SVTs), together with long-term follow-up.

## 2. Materials and Methods

### 2.1. Study Population

This was a single-center, retrospective observational study. Patients with symptomatic, documented supraventricular tachycardia (SVT) who underwent cardiac ablation between January 2020 and August 2024 at University Hospital Center Zagreb were included. Patients with atrioventricular nodal reentrant tachycardia (AVNRT), atrioventricular reentrant tachycardia (AVRT), atrial tachycardia (AT), and atrial flutter (AFL) were included. Patients with preexcitation in electrocardiogram (ECG) and medical history of palpitations were also included whereas patients with atrial fibrillation (AF) were excluded. A total of 400 patients were included, divided into two cohorts. The first consisted of 200 consecutive patients in whom ablation was completed without the use of fluoroscopy, utilizing mapping technology together with ICE. The second group consisted of 200 consecutive patients in whom ablation was completed under fluoroscopy guidance. The patients were allocated according to the operator’s discretion. The information was collected from the hospital’s electronic database as well as through interactions with patients. All procedures were carried out by skilled operators. All patients provided written informed consent for the ablation and the research was conducted in accordance with the principles of the Declaration of Helsinki.

### 2.2. Ablation Protocol

**Figure 1:**
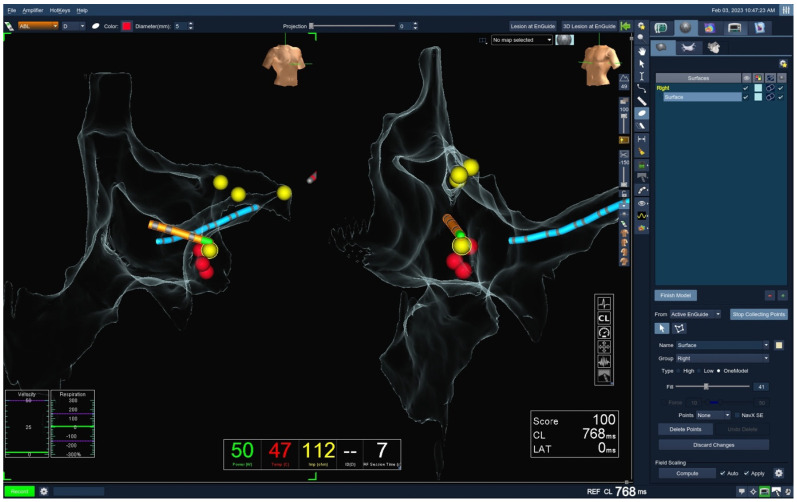
Mapping during procedure (Ensite). The reconstruction of the right atrium (RA), superior vena cava (SVC), and inferior vena cava (IVC) can be seen with the decapolar catheter (blue) placed in the coronary sinus (CS). The ablation catheter (orange) is also visualized with ablation lesions (red). RA, right atrium; SVC, superior vena cava; IVC, inferior vena cava; CS, coronary sinus.

All procedures were conducted with conscious sedation, using fentanyl and midazolam. Femoral vascular access was obtained under ultrasound guidance according to the operator’s discretion. For the fluoroless group, Ensite Precision (Abbott, St. Paul, MN, USA) or CARTO 3 (Biosense Webster, Diamond Bar, CA, USA) mapping systems were used to construct electroanatomic maps of the right atrium (RA), superior vena cava (SVC), inferior vena cava (IVC), His bundle, coronary sinus (CS) ostium, and tricuspid valve (TV). Furthermore, a decapolar catheter was placed in the coronary sinus and a quadripolar catheter in the right ventricle (Figure 1). After positioning all diagnostic catheters, a standard electrophysiology (EP) study was performed. For the control group, the procedure was performed under fluoroscopy guidance.

**Figure 2:**
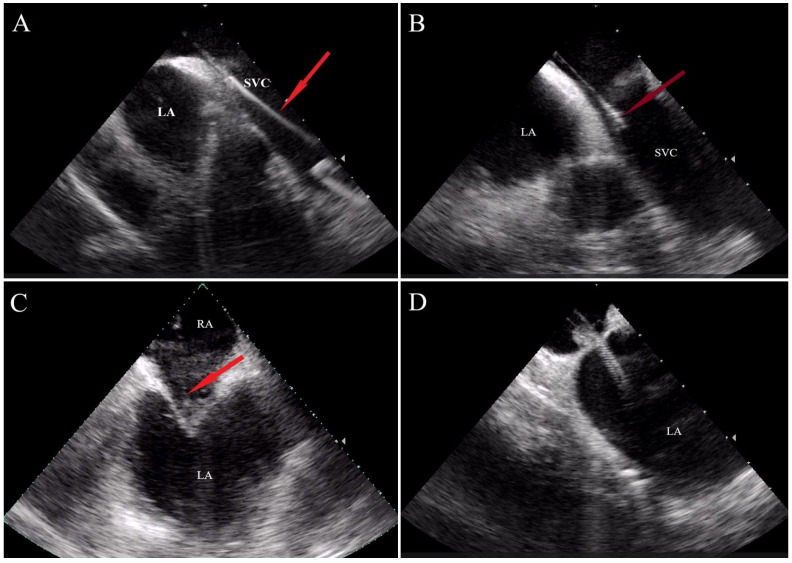
Transseptal puncture (TSP) with ICE. (**A**) The guidewire and long sheath (red arrow) are positioned in the superior vena cava (SVC). (**B**) The sheath is gradually pulled downward (red arrow) toward the fossa ovalis, initiating the “tenting” phase of the transseptal puncture (red arrow). (**C**,**D**) Following successful transseptal puncture, the sheath is visualized within the left atrium (LA). SVC, superior vena cava; LA, left atrium; RA, right atrium.

For ablation, radiofrequency catheters were used in the majority of patients, except in cases with a higher risk of conduction system damage—such as proximal His bundle or impending total AV block—where cryoablation using a focal cryo catheter (Freezor, Medtronic, Fridley, MN, USA) was performed. In cases with left-sided accessory pathways, atypical left-sided atrial tachycardia (AT), or atrial flutter (AFL), a left-sided approach was obtained by transseptal puncture (TSP) under ICE-guidance in both groups (Figure 2). An additional vascular access was obtained for the ICE catheter (Acuson AcuNav phased array catheter, Siemens AG). Additionally, for right-sided ablation procedures ICE was used in certain CTI ablation cases where lesion formation was more difficult to achieve.

A bolus of unfractionated heparin (100–150 IU/kg) was administered promptly after transseptal puncture. Activated clotting time (ACT) was checked every 10 to 15 min, and additional UFH boluses were provided as needed to maintain a target ACT above 300 s. Following TSP, further mapping and ablation were performed. After ablation, standard tachycardia inducibility testing was performed. Tachycardia non-inducibility for AVNRT, AVRT, AT, and atypical AFL was tested with and without isoproterenol challenge. For typical atrial flutter (AFL), after cavotricuspid isthmus (CTI) ablation, persistent bidirectional block was observed. In cases of TSP, while extracting the ICE catheter, the presence of pericardial effusion was checked, and all patients in whom TSP was performed underwent transthoracic echocardiography prior to discharge.

### 2.3. Follow-Up

Following hospital discharge, patients were assigned follow-up appointments at three and six months, during which a physical examination and 12-lead ECG were performed. In cases of palpitations, a 24-h Holter monitor was obtained. In addition, follow-up phone calls were conducted with patients. Any SVT episodes recorded after the initial procedure were classified as recurrences. Other modalities of ECG monitoring, such as smartwatches, trans-telephonic monitoring, or ECG patches, were not available for this study.

### 2.4. Endpoints

The primary endpoints were defined as feasibility—the possibility of completing the procedure without fluoroscopy in the main group (ZF)—and safety, defined as major complications related to the procedure, including cardiac tamponade, arteriovenous (AV) fistula, persistent total AV block, atrioesophageal (AE) fistula and systemic thromboembolism in both the main (ZF) and control groups. Secondary endpoints included (i) procedural parameters such as procedure duration and ablation time, radiation dose in the control group, etc.; (ii) acute success—arrhythmia non-inducibility in both groups; and (iii) long-term outcomes—freedom from arrhythmia recurrence during follow-up for both groups.

### 2.5. Statistical Analysis

The normal distribution was tested using the Shapiro-Wilk test. Continuous variables followed a normal distribution and are therefore presented as mean ± standard deviation. Categorical variables are described as absolute values and percentages. Comparisons between groups were undertaken with parametric tests for continuous variables (Student’s t-test) and non-parametric (chi-square test) tests for categorical variables. The Cox proportional hazards model was used to compare the association between the ablation approach and arrhythmia recurrence during the follow-up period. A *p*-value < 0.05 was deemed statistically significant. Statistical analyses were conducted using the JASP program (JASP 0.95.2).

## 3. Results

### 3.1. Patient Characteristics

A total of 400 patients were included, with 200 in the main (ZF) group and 200 in the control (fluoroscopy) group. There was no statistical difference in baseline characteristics between the groups regarding age (50.9 ± 16.5 years vs. 51.9 ± 16.1 years, *p* = 0.652), body mass index (26.7 ± 4.8 vs. 27.5 ± 6.0, *p* = 0.507), and left ventricular ejection fraction (57.6 ± 10.9% vs. 56.9 ± 11.8%). The proportion of male patients was lower in the main group (47.5% vs. 58.5%, *p* = 0.031) and there were fewer patients with an implanted pacemaker (PM) or implantable cardioverter–defibrillator (ICD) device (1.5% vs. 4.5%, *p* = 0.010). With respect to antiarrhythmic drugs (AADs), patients were most commonly on beta-blockers (51.5% vs. 55.0%, *p* = 0.470). Detailed baseline clinical characteristics are provided in Table 1.

### 3.2. Procedural Characteristics

The most common indication for ablation was AVNRT in both groups (56.0% vs. 50.0%, *p* = 0.232), followed by AFL (22.0% vs. 33.5%, *p* = 0.012), AVRT (16.5% vs. 15.5%, *p* = 0.425), and AT (5.5% vs. 1.0%, *p* = 0.012). Procedures were shorter in the main group (59.0 ± 25.8 min vs. 72.7 ± 34.0 min, *p* < 0.001) with similar ablation duration (418.7 ± 898.8 s vs. 558.4 ± 646.6 s, *p* = 0.075). Acute success was achieved in all patients (100% vs. 100%, *p* = 0.152). Detailed procedural characteristics are provided in Table 2.

### 3.3. Complications and Outcomes

During a six-month follow-up, there was no statistical difference regarding arrhythmia recurrence in the fluoroless compared to the fluoroscopy group (6.0% vs. 5%, *p* = 0.670), with a hazard ratio of 0.877 (95% CI, 0.367–2.097, *p* = 0.769). Regarding serious complications, there was one case of persistent AV block in the control group that required permanent pacemaker implantation (*p* = 0.313). (Table 3).

## 4. Discussion

The main findings of the study are: (a) a zero-fluoro, apron-less approach to SVT ablation is feasible; (b) the fluoroless approach has an excellent safety profile, as no serious complications were registered; and (c) arrhythmia recurrence following fluoroless ablation is acceptable and non-inferior to conventional ablation.

In interventional cardiology, including interventional electrophysiology (EP), fluoroscopy has traditionally been used as the visualization modality for procedures. Therefore, ionizing radiation in the EP laboratory exposes staff to occupational radiation exposure [13,14]. Furthermore, the use of leaded aprons to mitigate radiation exposure can lead to orthopaedic complications [15]. Overall, interventional electrophysiologists have a 10% higher risk of radiation-related illness and more than a 50% higher risk of orthopaedic injury compared to other cardiologists [16]. As a result, the “as low as reasonably achievable” (ALARA) principle has been adopted to reduce radiation exposure and its complications for both staff and patients. Therefore, the prospect of performing the procedures with no fluoroscopy not only eliminates the potential complications of radiation exposure, but also removes the need for wearing X-ray protective gear- hence the term “apron-less” approach. In addition to providing greater comfort, this technique also eliminates the significant orthopaedic risks commonly faced in electrophysiology [17].

The development and advancement of mapping systems have enabled a significant reduction in the use of fluoroscopy in interventional EP [18,19]. These systems also offer more precise localization of arrhythmia mechanisms and enable catheter positioning relative to adjacent structures such as the His bundle, potentially reducing complications. Recent advancements in magnet-based electroanatomic mapping systems have further enabled mapping and ablation of left-sided SVTs, such as atypical atrial flutter or left-sided accessory pathways. For these procedures, the use of intracardiac echocardiography (ICE) has been shown to be pivotal, as it enables left-sided access via transseptal puncture (TSP) without the use of fluoroscopy.

In our study, acute success was achieved in all patients in both the main (ZF) and control (fluoroscopy) groups. There was no difference in arrhythmia recurrence during the six-month follow-up (HR: 0.877, 95% CI: 0.367–2.097, *p* = 0.769), with the majority of patients free from arrhythmia recurrence (94.0% vs. 95.0%, *p* = 0.670) and there were no major complications in the main (ZF) group, compared to one case of persistent AV block requiring pacemaker implantation in the control group (*p* = 0.313). In the main (fluoroless) group, there were seven cases where cryoablation was performed, with a slightly higher recurrence rate compared to RF ablation procedures, but without statistical significance (14.2% vs. 5.7%, *p* = 0.345) and with no serious complications. Procedures were shorter in the main (ZF) group (59.0 ± 25.8 min vs. 72.7 ± 34.0 min, *p* < 0.001). Although this difference is not necessarily clinically significant, it might permit more streamlined patient scheduling in high-volume electrophysiology (EP) labs.

Our data are consistent with current literature. Bergonti et al. [20] reported that the minimal fluoroscopy approach is feasible in a group of 206 patients with good long-term outcomes (median follow-up 4.2 years), and better arrhythmia-free survival compared to the traditional fluoroscopy ablation group of 412 patients (97.4% vs. 91.1%). Giaccardi et al. [21] reported outcomes of completely fluoroless ablation in a group of 266 patients. Their findings match ours with regard to acute success (100%) and complication rates (one case of pericardial tamponade). They achieved chronic success in 90.8% of patients during follow-up (2.9 ± 1.6 years). In cases where cryoablation was performed for AVRT, they found higher recurrence rates (*p* = 0.001). Prolič Kalinšek et al. [22] reported outcomes of completely fluoroless ablation in a group of 294 patients compared to conventional fluoroscopy-based ablation in a group of 280 patients. Notably, they reported a statistically significant reduction in procedure time in their fluoroless group (94.2 ± 50.4 min vs. 104.0 ± 54.0 min, *p* < 0.001), which is consistent with our findings, as we also found procedure duration to be shorter in the main (ZF) group compared to the control group (59.0 ± 25.8 min vs. 72.7 ± 34.0 min, *p* < 0.001). Furthermore, they found ablation time to be significantly longer in the fluoroless (ZF) group compared to the fluoroscopy group (382.0 ± 379.0 s vs. 233.0 ± 242.0 s, *p* < 0.001), which differs from our findings where ablation time was shorter in the main (ZF) group, but without statistical significance (418.7 ± 898.8 s vs. 558.4 ± 646.6 s, *p* = 0.075). As for complications, no major complications occurred in either group. Overall success rate during follow-up (424 ± 338 days) was higher in the fluoroless (ZF) group (98.3% vs. 93.5%, *p* = 0.004).

Authors from China [23] reported a prospective multicenter study in which 1020 patients underwent ablation without the use of fluoroscopy, and 2040 patients underwent ablation with fluoroscopy. They reported similar acute success rates (98.8% vs. 99.2%, *p* = 0.22) with low recurrence rates during a 12-month follow-up (0.4% vs. 0.5%, *p* = 0.85).

A meta-analysis [12] comparing the M/ZF to the conventional fluoroscopy approach including 24 studies involving 9074 patients reported no difference in acute success (97.4% vs. 97.55%; RR: 1.0, 95% CI: 0.99–1.01, *p* = 0.97) and long-term success rate (97.02% vs. 96.17%; RR: 1.01, 95% CI: 1.00–1.03, *p* = 0.13), with a mean length of the follow-up that varied between 42 and 1584 days. For procedural parameters, ablation time was shorter in the Z/MF method (MD: −2.53 s (95% CI: −42.04 to −8.43 s, *p* < 0.01) with no difference in the total procedural time (MD: 3.06 min (95% CI: −0.97 to 7.08 min; *p* = 0.14). In our study we found the procedure duration to be shorter in the fluoroless group, and although this differs from the results of the reported meta-analysis, it is highlighted that there was a significant heterogeneity in the included studies, possibly because of the broad type of arrhythmia ablations being performed. Our level of acute and chronic success rates is similar and we also found the level of complications to be very low. It is also important to emphasize that only 4 of the studies included in the meta-analysis report a completely fluoroless approach achieved in all cases.

Notably, fluoroless ablation was achieved in patients with higher complication potential, such as those with implanted pacemaker devices. In our study, all patients with implanted devices who underwent ablation without fluoroscopy had no serious complications related to ablation or implanted pacemakers, such as lead dislocation, and had good results during follow-up that did not differ from other patients. The use of intracardiac echocardiography (ICE) allows for direct visualization of the ablation substrate and catheter movement, which not only reduces the risk of complications but also facilitates the delivery of adequate ablation lesions in challenging cases. In this study, ICE was primarily employed to enable fluoroless transseptal puncture (TSP) and in certain typical atrial flutter (AFL) ablation procedures where bidirectional block was not achieved despite extensive ablation. Additionally, the use of ICE in supraventricular tachycardia (SVT) ablation offers further potential benefits, such as reduced procedure and ablation times for atrioventricular nodal reentrant tachycardia (AVNRT) [24]. Several studies have demonstrated that cavotricuspid isthmus (CTI) ablations can be performed safely and effectively under exclusive ICE guidance, without the need for electroanatomical mapping systems (EAMS) or fluoroscopy [25,26]. These findings underscore the ability of ICE to facilitate the formation of complete CTI ablation lines without compromising safety or prolonging procedure duration. ICE is particularly valuable in cases involving extended CTI isthmus lengths or anatomical variants where more extensive ablation is required.

Since the widespread use of ICE catheters is limited due to significant financial constraints and their single-use design, ICE catheter reprocessing can be considered, as data show feasibility and safety of this method [27]. Regarding cases where cryoablation was used, our data show no difference compared to RF procedures, which is in contrast with current literature [28]. However, some studies [22] also report outcomes following cryoablation comparable to those conducted with RF ablation.

Our data encourage the fluoroless, “apron-less” approach for the ablation of supraventricular tachycardia. The advancement in electroanatomic mapping systems has made this achievable, and together with ICE it enables these procedures to be carried out for a broad range of SVTs with additional potential for reducing complications. Moreover, eliminating fluoroscopy offers health benefits for both staff and patients [29].

## 5. Conclusions

Our study suggests that completely fluoroless ablation of SVT is feasible, safe, and associated with good clinical outcomes compared to the conventional fluoroscopy-guided approach.

## 6. Limitations

This was a retrospective, single-center study with all inherent limitations of this type of research. Ideally, a randomized trial is needed to draw definitive conclusions. We did not detect any major complications in our cohort, which is promising, but it is likely that major complications would have been observed in a larger cohort. Intracardiac echo catheters are not widely available globally due to reimbursement issues. This, together with the fact that EAMs represent a financial burden, is an important limitation that may prevent widespread adoption of our strategy. Furthermore, our follow-up period was six months; a longer follow-up would provide a clearer view of the durability of the zero-fluoro approach. Also, ECG and Holter monitoring were the only diagnostic tools available which could lead to recurrence under-detection that could potentially bias the results. Therefore, we also conducted interviews with the patients and symptomatic typical episodes were considered recurrences in order to minimize the potential recurrence under-detection.

## Figures and Tables

**Table 1:** Baseline characteristics. BMI body mass index, COPD chronic obstructive pulmonary disease, HFrEF heart failure with reduced ejection fraction, OSAS obstructive sleep apnea syndrome, PM pacemaker, ICD implantable cardioverter–defibrillator, AADs antiarrhythmic drugs, LVEF left ventricular ejection fraction, VKA vitamin K antagonist, DOAC direct oral anticoagulants.

Characteristic	Fluoroless	Control	*p* Value
Age, years (mean ± SD)	50.9 ± 16.5	51.9 ± 16.1	0.540
Gender, male, n (%)	95 (47.5)	117 (58.5)	0.031
BMI (mean ± SD)	26.7 ± 4.8	27.5 ± 6.0	0.142
Arterial hypertension, n (%)	80 (40.0)	90 (45.0)	0.319
Diabetes mellitus, n (%)	18 (9.0)	19 (9.5)	0.851
Coronary artery disease, n (%)	9 (4.5)	13 (6.5)	0.367
Chronic kidney disease, n (%)	8 (4.0)	7 (3.5)	0.808
COPD, n (%)	4 (2.0)	8 (4.0)	0.238
Hyperlipidemia, n (%)	58 (29.0)	58 (29.0)	0.949
HFrEF, n (%)	11 (5.5)	14 (7.0)	0.426
OSAS, n (%)	2 (1.0)	5 (2.5)	0.250
Valvular heart disease, n (%)	11 (5.5)	13 (6.5)	0.646
PM/ICD implanted, n (%)	3 (1.5)	9 (4.5)	0.010
AADs, n (%)	I 28 (14.0)	I 27 (13.5)	0.900
	II 103 (51.5)	II 110 (55.0)	0.470
	III 14 (7.0)	III 22 (11.0)	0.280
	IV 8 (4.0)	IV 4 (2.0)	0.245
Anticoagulation, n (%)	VKA 6 (3.0)	VKA 12 (6.0)	0.148
	DOAC 49 (24.5)	DOAC 51 (25.5)	0.885
LVEF, % (mean ± SD)	57.6 ± 10.9	56.9 ± 11.8	0.538

**Table 2:** Procedural characteristics. AVNRT atrioventricular nodal reentrant tachycardia, AVRT atrioventricular reentrant tachycardia, AT atrial tachycardia, AFL atrial flutter.

Characteristic	Fluoroless	Control	*p* Value
Indication, n (%)	AVNRT 112 (56.0)	AVNRT 100 (50.0)	0.232
	AVRT 33 (16.5)	AVRT 31 (15.5)	0.425
	AT 11 (5.5)	AT 2 (1.0)	0.012
	AFL 44 (22.0)	AFL 67 (33.5)	0.012
Duration, min (mean ± SD)	59.0 ± 25.8	72.7 ± 34.0	<0.001
Fluoroscopy duration, min (mean ± SD)	0	11.4 ± 9.5	<0.001
Fluoroscopy total, mGy (mean ± SD)	0	19.6 ± 26.9	<0.001
Fluoroscopy dose, Gy/cm^2^ (mean ± SD)	0	174.8 ± 252.9	<0.001
Ablation duration, s (mean ± SD)	418.7 ± 898.8	558.4 ± 646.6	0.075
Acute success, n (%)	200 (100.0)	200 (100.0)	0.152
TSP, n (%)	32 (16.0)	21 (10.5)	0.200

**Table 3:** Outcomes. AV-block atrioventricular block.

	Fluoroless	Control	*p* Value
Arrhythmia recurrence, n (%)	12 (6.0)	10 (5.0)	0.670
Time to recurrence, days (mean ± SD)	127.5 ± 41.4	140.0 ± 24.0	0.410
Complications, n (%)	0 (0)	Total AV-block 1 (0.5)	0.313

## Data Availability

The datasets presented in this article are not readily available because of Croatian legal regulations. Data are, however, available from the corresponding author upon reasonable request and with permission from KBC Zagreb.

## Abbreviations

The following abbreviations are used in this manuscript:

3D	Three dimensional
AADs	Antiarrhythmic drugs
ACT	Activated clotting time
AE	Atrioesophageal
AF	Atrial fibrillation
AFL	Atrial flutter
ALARA	As low as reasonably achievable
AT	Atrial tachycardia
AV	Arteriovenous
AV	Atrioventricular
AVNRT	Atrioventricular nodal reentrant tachycardia
AVRT	Atrioventricular reentrant tachycardia
BMI	Body mass index
COPD	Chronic obstructive pulmonary disease
CS	Coronary sinus
CTI	Cavotricuspid isthmus
DOAC	Direct oral anticoagulants
EAMS	Electroanatomical mapping systems
ECG	Electrocardiogram
EP	Electrophysiology
HFrEF	Heart failure with reduced ejection fraction
ICD	Implantable cardioverter–defibrillator
ICE	Intracardiac echocardiography
IVC	Inferior vena cava
LA	Left atrium
LVEF	Left ventricular ejection fraction
MF	Minimal fluoroscopy
OSAS	Obstructive sleep apnea syndrome
PM	Pacemaker
RA	Right atrium
RF	Radiofrequency
SVC	Superior vena cava
SVT	Supraventricular tachycardia
SVTs	Supraventricular tachycardias
TSP	Transseptal puncture
TV	Tricuspid valve
UFH	Unfractionated heparin
VKA	Vitamin K antagonist
ZF	Zero fluoroscopy
